# Immediate dental implant placement: A bibliometric analysis of the 100 most cited articles

**DOI:** 10.4317/jced.62274

**Published:** 2025-05-01

**Authors:** Lucas Menezes dos Anjos, Aurélio de Oliveira Rocha, Felipe Gomes Dallepiane, Fernanda Pretto Zatt, Filipe Colombo Vitali, Pablo Silveira Santos, Christiane Cabral Leite, Natalia de Oliveira Miranda, Ariadne Cristiane Cabral Cruz, Mariane Cardoso, Bruno Henriques

**Affiliations:** 1 Post-Graduation Program in Dentistry, Federal University of Santa Catarina, Florianópolis, Santa Catarina, Brazil; 2 Department of Mechanical Engineering, Federal University of Santa Catarina, Florianópolis, Santa Catarina, Brazil

## Abstract

**Background:**

This study aims to scrutinize the characteristics of the 100 most cited articles concerning the immediate placement of dental implants.

**Material and Methods:**

Employing a specific search strategy comprising keywords and Boolean operators, we conducted a comprehensive search in the Web of Science database in January 2024. The identified articles were then arranged in descending order based on their citation counts. Two researchers meticulously gathered information on the number and density of citations, publication year and journal, study design, authorship and affiliations, keywords, as well as the country and continent of origin. Bibliometric maps were constructed using VOSviewer.

**Results:**

The citations for the selected articles ranged from 76 to 569, spanning the publication years 1994 to 2018. Clinical Oral Implants Research emerged as the most frequently referenced journal, accounting for 30% of the publications. Interventional studies constituted the majority (29%) of the articles. Buser D and Lang NP were the most prolific authors, each contributing 11% of the published articles. The University of Bern had a total representation of 10%. The United States garnered significant attention at 20%, while Europe emerged as the predominant continent, constituting 46% of the publications. VOSviewer maps illustrated a convergence of authorship.

**Conclusions:**

The 100 most cited articles on immediate implant placement predominantly originated from authors in the United States, emphasizing Clinical Oral Implants Research as a notable outlet featuring observational studies.

** Key words:**Immediate implantation, Bibliometric analysis, Dental implants.

## Introduction

The concept of osseointegration, developed and introduced by Dr. Brånemark, describes the direct structural and functional connection between living bone and the surface of an implant, which is essential for ensuring its stability, while also establishing a protocol for dental implant placement. He recommended a six-month healing period after tooth extractions to allow proper recovery of the alveolar bone before implant installation. This healing process was shown to enhance osseointegration, ultimately improving the predictability and success of the implants (1,2). Subsequent studies have indicated that dental implants can be immediately installed in the alveolar bone remnant following extractions, a technique known as immediate implant installation (3).

Nevertheless, specific requirements must be considered before conducting this technique. These include the absence of periapical infections, sufficient bone quantity for implant anchorage, and a minimally invasive extraction technique that preserves the maximum amount of bone remnant, along with the operator’s experience (3,4). The protocol for immediate implant installation is considered a predicTable surgical technique, offering advantages such as reducing surgical procedures and performing a less invasive surgical procedure by not requiring flap elevation, which also ensures better preservation of soft tissues surrounding the implants (5).

Bibliometric analysis uses quantitative tools based on data from published studies to identify and reveal scientific trends in a specific clinical field. Among the metrics involved in bibliometric analysis, the number of citations an article receives can be deemed a significant indicator of the relevance and merit of studies and scientific journals. Highly cited studies might be considered classics in a particular field of knowledge (6). Bibliometric analyses can also assist researchers in identifying key author groups, institutions, countries, themes, study designs, among other characteristics within a specific area (7).

Subjects in dental implant have been covered in previous bibliometric studies (8-13). However, to our knowledge, no scientific literature has analyzed the scientific profile of publications regarding immediate dental implant installation. Such a study could assist researchers in identifying the main themes and research trends related to this technique in dental implant. Moreover, this understanding can help identify the principal gaps in the scientific knowledge, thereby encouraging further studies in this area. Therefore, this bibliometric study aimed to analyze the characteristics of the top 100 most-cited articles related to immediate dental implant installation.

## Material and Methods

-Information sources and search strategy

The primary search was conducted on January 07, 2024, employing a qualitative-quantitative approach, in the Web of Science Core Collection (WoS-CC) database. This database was selected as it is widely used in the scientific field and enables the articles to be sorted in descending order based on the number of citations. For article selection, a search strategy was devised by combining keywords using Boolean operators [ALL= (“immediate dental implant* placement*” OR “immediate implant* placement*” OR “immediate implant*” OR “immediate implant* placement*” OR “dental implant* early” OR “early implant* placement*” OR “dental implant* early” OR “early implant* placement”)].

-Eligibility criteria

Articles that assessed the theme ‘immediate dental implant installation’ were included without any restrictions on publication date or language. Studies not related to the investigated topic were excluded from the analysis.

-Selection process and data cross-referencing

The primary search provided a list of articles within the WoS-CC database, arranged by the number of citations in descending order. The 100 most-cited articles were identified by two independent researchers (LMA and AOR) through reviewing titles, abstracts, and full texts when necessary. Discrepancies were resolved through consensus with a third researcher (BH).

-Extraction of data from selected studies

The following bibliometric parameters were gathered for each article: title, authors, number of authors, number of citations, institution, country, continent (based on the corresponding author’s affiliation), publication year, journal title, journal’s impact factor in 2023 (when identified in the Journal Citation Reports), keywords, and study design. The study designs were categorized as follows: systematic reviews, literature reviews, laboratory studies, observational studies, clinical case reports or case series, and intervention studies (clinical trials). All articles were grouped into the following topics taking into account their objectives: use of bone substitute, surgical complications, use of connective graft, evaluation of the dimension of the alveolar bone, technique description, evaluation of effectiveness, association with provisory prosthesis, use of guided bone regeneration, patient satisfaction assessment, and immediate implants at infected sites.

-Bibliometric and statistical analysis of the data

The Visualization of Similarities Viewer (VOSviewer, Leiden University, Netherlands) software was utilized to generate a graphical representation of bibliometric networks, illustrating the connections among authors, countries, and keywords. The size of the circles is proportional to the intensity of data in the network. Items represented by similar colors and interconnected in the same cluster demonstrate the correlation between the data. For data analysis, the statistical software package SPSS for Windows (SPSS, version 24.0; IBM Corp) was used. Normality was checked using the Kolmogorov-Smirnov test. The Spearman’s correlation coefficient test was employed to verify the correlation between the number of citations and the publication year, as the data did not follow a normal distribution.

## Results

-Research results

Based on the established strategy, the initial search yielded 2,487 documents, listed in descending order by the number of citations. To select the top 100 most-cited studies, the first 190 documents were analyzed. The 90 excluded studies were not related to the objective proposed in the study. This resulted in the identification of the top 100 most-cited articles on immediate implant placement. These articles can be requested directly from the corresponding author

-Analysis of the number of citations

The top 100 most-cited articles totaled 16,063 citations in the WoS-CC database, with an average of 160 citations per article. Among these, 705 (4.4%) were self-citations. The number of citations ranged from 76 to 569, with 74% of the articles cited at least 100 times. The most-cited article was “Hard-tissue alterations following immediate implant placement in extraction sites” an interventional study authored by Botticelli D, Berglundh T, and Lindhe J in Sweden, published in 2004 in the Journal of Clinical Periodontology.

-Year of publication

**Figure 1:**
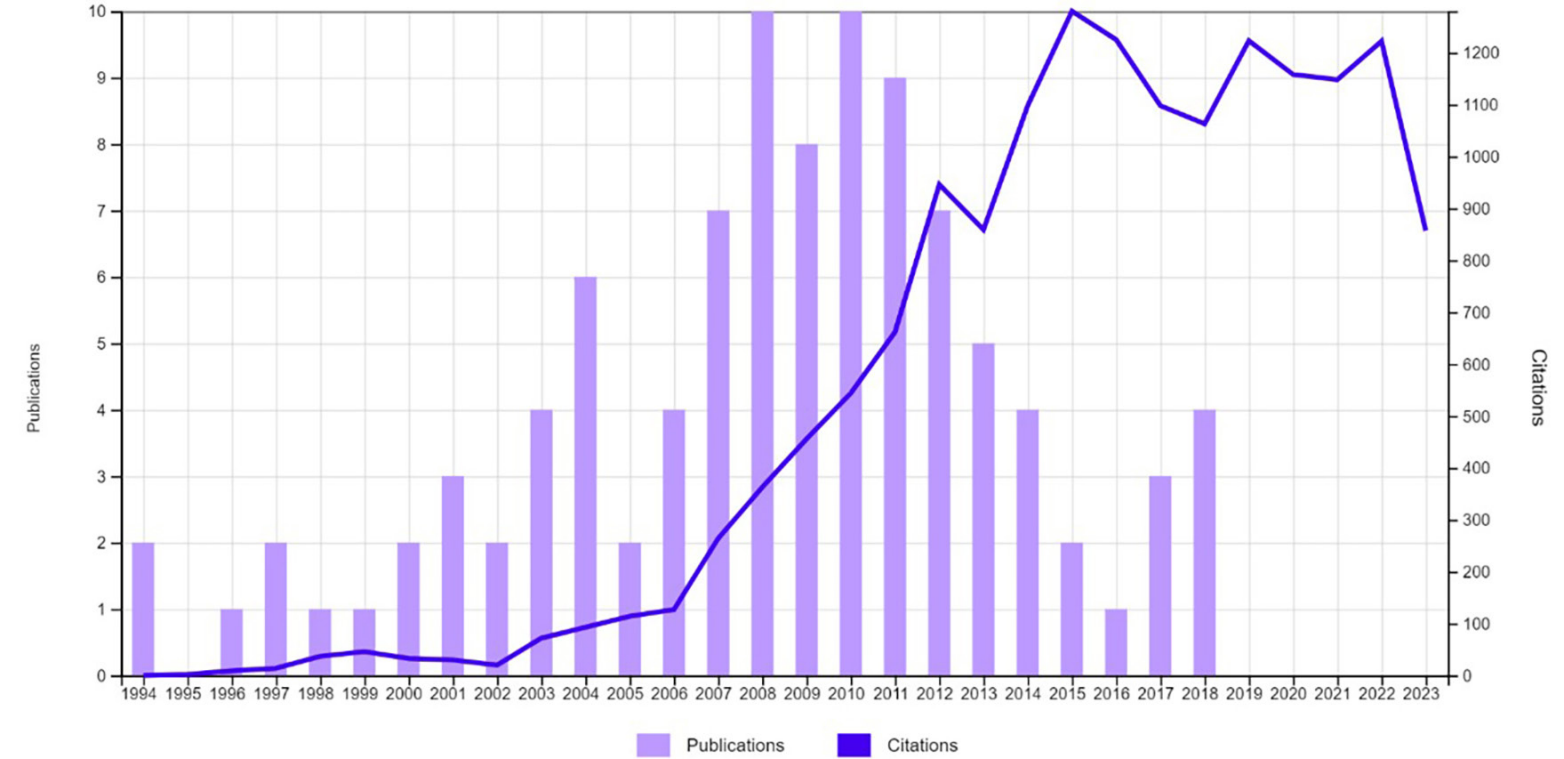
Distribution of the number of publications over the years.

Published in June 1994 the oldest article was: “immediate implant placement using a biodegradable barrier, polyhydroxybutyrate-hydroxyvalerate reinforced with polygalactin-910 - an experimental-study in dogs” written by Gotfredsen K and collaborators, published in Clinical Oral Implants Research. Published in October 2018, the most recent article was: “Implant placement and loading protocols in partially edentulous patients: A systematic review” written by Galluci GO and collaborators, published in Clinical Oral Implants Research. Among the 100 most-cited articles on immediate implant placement, the period between 2004 and 2013 holds the highest number of publications (68 articles; 11,485 citations) as depicted in Figure 1. According to Spearman’s correlation, a weak negative correlation was observed between the number of citations and year of publication (rho = −.162; *p-value* >0.05).

-Contributing journals and impact factor

As indicated in  Table 1, the Clinical Oral Implants Research stands out as the journal with the most publications in this top 100 list (30 articles; 5,172 citations), followed by the International Journal of Oral & Maxillofacial Implants (23 articles; 3,892 citations) and the Journal of Periodontology (18 articles; 2,882 citations). According to the Journal Citation Reports, the journals with the highest impact factor (IF) in 2023 related to immediate implants were: Periodontology 2000 with one study (IF 17.5; 104 citations), Cochrane Database of Systematic Reviews with one study (IF 8.8; 110 citations), and Journal of Clinical Periodontology with two studies (IF 5.8; 1,862 citations).

-Theme and Study design

Among of the top 100 most-cited studies, the majority are interventional (29 articles; 4,214 citations), followed by observational (24 articles; 3,940 citations), case report (15 articles; 2,219 citations), literature reviews (11 articles; 2,292 citations), laboratory studies (11 articles; 1,680 citations), and systematic reviews (10 articles; 1,718 citations). Considering the topic related to the objective of the included articles, most studies evaluated the effectiveness of immediate implant installation (53 articles; 3,944 citations), followed by technique description (12 articles; 2,255 citations), association with provisory prosthesis (8 articles; 1,040 citations), evaluation of the dimension of the alveolar bone (7 articles; 1,123 citations), use of guided bone regeneration (5 articles; 649 citations), immediate implants at infected sites (5 articles; 544 citations), use of connective graft (4 articles; 396 citations), surgical complications (3 articles; 407 citations), use of bone substitute (2 articles; 307 citations) and just one article evaluated the patient satisfaction assessment (1 articles; 78 citation).

-Countries and continents and Financing agency

**Figure 2:**
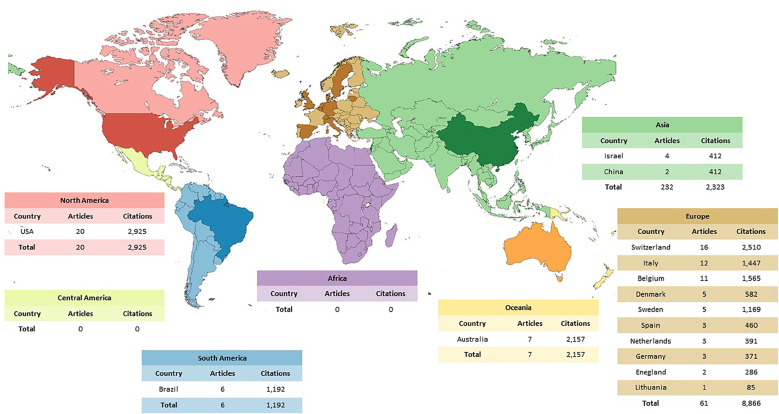
Worldwide distribution of the origin of publications on immediate placement of dental implants. The continents are outlined in lighter shades and the countries associated with the articles are identified with darker shades referring to their continent.

As depictable in Figure 2, among the continents represented in the 100 most-cited articles, Europe stands out with 61 articles and 8,866 citations, followed by North America (20 articles; 2,925 citations), Oceania (7 articles; 2,157 citations), South America (6 article; 1,192 citations), and Asia (6 articles; 923 citations). In terms of the number of publications per country, the United States leads with 20 articles totaling 2,925 citations in the WoS-CC, followed by Switzerland (16 articles; 2,510 citations), Italy (12 articles; 1,447 citations), Belgium (11 articles; 1,565 citations), Australia (7 articles; 2,157 citations), Brazil (6 articles; 1,192 citations), Denmark (5 articles; 582 citations), Sweden (5 articles; 1,169 citations), Israel (4 articles; 511 citations), Spain (3 articles; 460 citations), Netherlands (3 articles; 391 citations), Germany (3 articles; 371 citations), England (2 articles; 286 citations), China (2 articles; 412 citations), and Lithuania with only one article (85 citations). A total of 4 funding agencies were linked to the publications included in this study, the main one being Astra Tech AB (3 articles, 545 citations), based in Sweden, followed by Nobel Biocare (1 article; 254 citations), Thommen Medical (1 article; 268 citations), and Osteology Foundation (1 article; 176 citations) both based in Switzerland.

-Institutions 

A total of 35 institutions were identified among the top 100 most-cited articles.  Table 2 highlights the five institutions with the most publications, particularly the University of Bern (10 articles; 1,371 citations), University of Loma Linda (8 articles; 1,236 citations), and University of Melbourne and University of Ghent with six articles each, and 1,691 and 787 citations, respectively.

-Keywords

**Figure 3:**
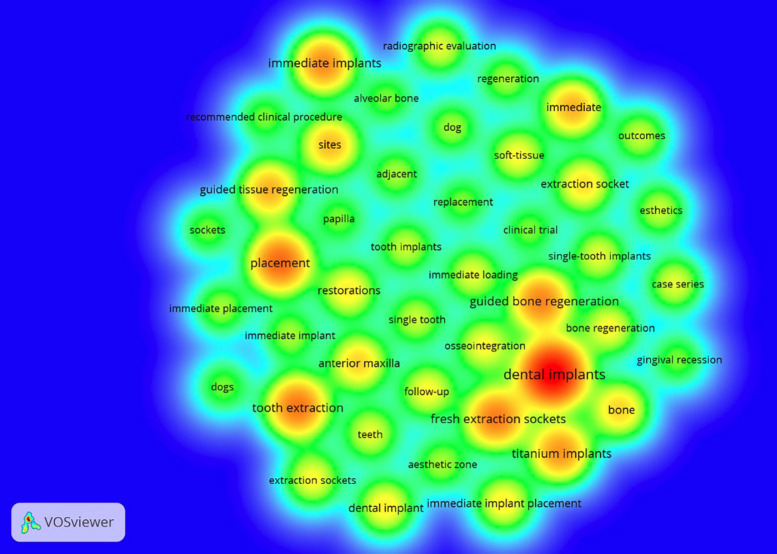
Map of keyword density among the 100 most-cited articles. The larger circles represent words with higher occurrences. On the contrary, words inside smaller circles had fewer occurrences. The largest circle the figure, in red, indicates that “dental implants” was the most frequently used keyword in the articles.

As for the keywords present in the top 100 most-cited articles, 504 different terms were identified, with emphasis on “dental implants” (47 occurrences), “placement” (26 occurrences), “tooth extraction” (24 occurrences), “fresh extraction sockets” (23 occurrences), and “guided bone regeneration” (22 occurrences each), as indicated in Figure 3.

-Authorship

**Figure 4:**
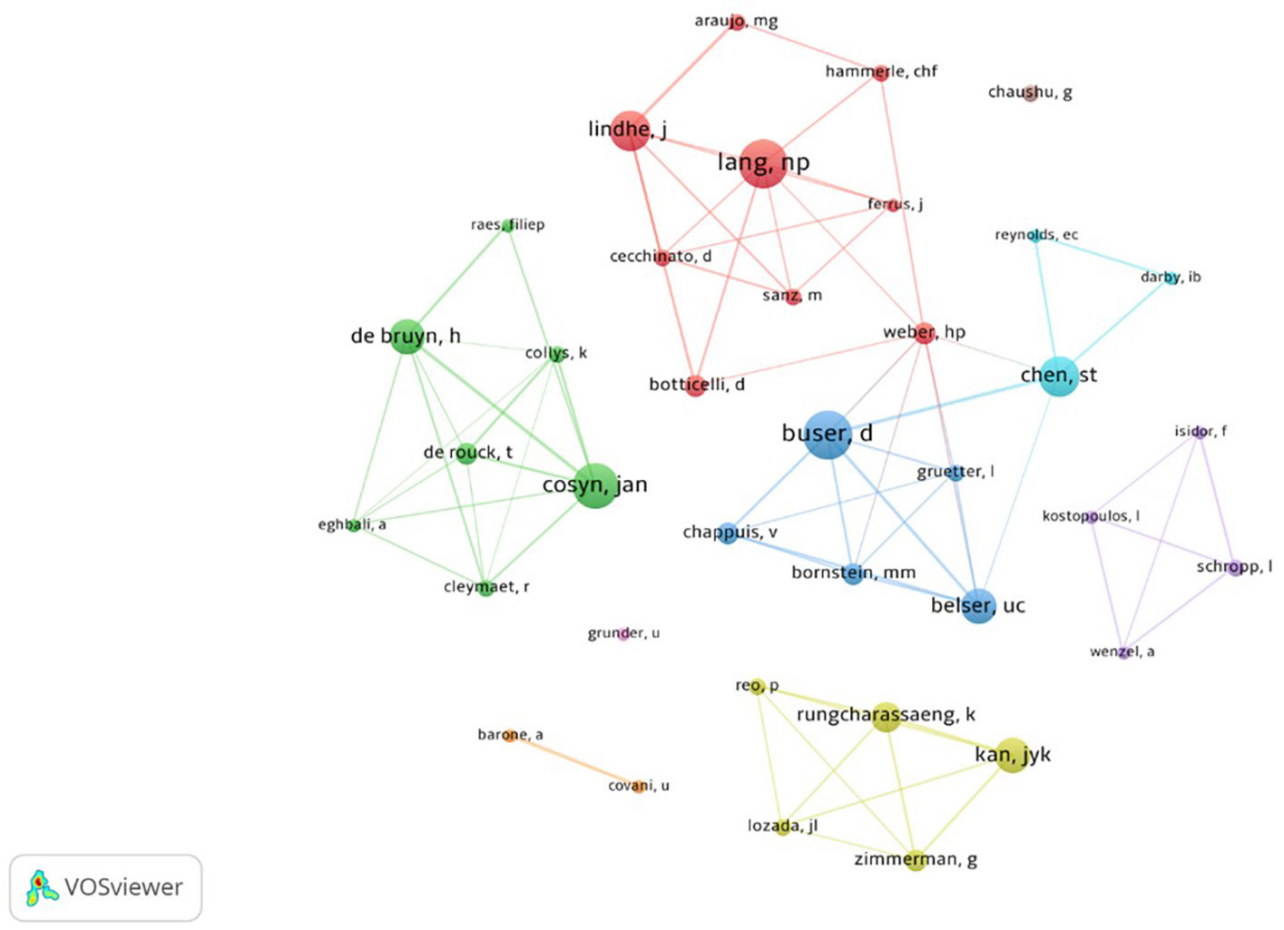
Map of author density and co-authorship among the 100 most-cited articles. Names highlighted in bold and corresponding to blue, red and green colored nodes are associated with authors of higher occurrence. Conversely, names associated with yellow and purple nodes correspond to authors with fewer occurrences. Moreover, the image illustrates the interrelation between author groups, showing isolated clusters in the center.

Figure 4 indicates a map of author frequency and co-author relationships among the 100 most-cited articles.  Table 3 displays the top five authors with the highest number of publications. Buser D and Lang NP were the authors with the most publications, eleven having six documents, accumulating 2,311 and 1,894 citations, respectively.

## Discussion

The present bibliometric review aimed to analyze the primary characteristics of the 100 most-cited articles related to the immediate installation of dental implants in the WoS-CC database. It was observed that the scientific advancement of this subject is primarily supported by interventional studies conducted in the European continent. According to the literature, this is the first bibliometric analysis that qualitatively and quantitatively evaluated the publications on immediate implant placement in dentistry.

The WoS-CC electronic database is a reference for conducting bibliometric studies as it allows for an analysis of research quality and impact (14). Web of Science enables the retrieval of current and retrospective multidisciplinary research. Moreover, it provides parameters and practical applications to evaluate a research team’s characteristics and the number of journal and article citations available through the Science Citation Index (15). As a result, many studies involving bibliometric analyses have used it as the primary search platform (6,16). Furthermore, within Web of Science, it is possible to sort studies based on their citation counts, making it easier and more secure to conduct searches. This is because the first articles in the list, sorted by a combination of keywords and Boolean operators, display the highest citation counts (16).

Of the 100 most-cited articles on immediate implant placement, 73 articles received a minimum of 100 citations. Studies that surpass 100 citations within a specific field can be considered classics in that area (17). Therefore, it is worth highlighting that the majority of selected articles exceed this number, meaning the significance of these contributions to the scientific progress on immediate dental implant placement. Additionally, only 4.4% of self-citations were identified. Despite self-citation not being favorably viewed, it may naturally occur due to the focused effort of specific authors in a particular field of knowledge (18).

The Clinical Oral Implants Research is a journal that conveys the scientific state on implantology and related areas focusing on manipulation and regeneration of peri-implant tissues. This journal stood out in this review by publishing the highest number of articles on immediate implant placement. It has also been prominent in other bibliometric analyses such as the one conducted by Tarazona *et al*. (19), where the authors performed a bibliometric review on scientific studies focused on implantology between 2009 and 2013. Additionally, the bibliometric analysis by Chiang *et al*. (20) evaluated publications primarily focusing on complications associated with implant-supported rehabilitations. The International Journal of Oral & Maxillofacial Implants was the second noteworthy journal in this review. This journal publishes basic or clinical research reports, clinical applications of research and implant technology, symposium or conference studies relevant to the field, review articles, and education issues related to implantology.

Between 2004 and 2013, the highest number of publications in this top 100 (68%) was concentrated, most likely due to a significant increase in publications involving dental implants during that period. Elani *et al*. (21) assessed the prevalence of dental implant use in the United States and identified a substantial increase in implant utilization between 1999 and 2016. According to the authors, the exponential rise in publications on dental implants during this time was due to excellent results and advancements in the osseointegration process, as well as an enhanced predictability in implant-supported rehabilitations. It was also during this period that the more effective use of biomaterials for bone augmentation and innovative surface treatments were more prominently proposed. This review suggests an association between increased dental implant use and the greater prevalence of studies during this period.

In addition to the favorable clinical outcomes already reported in the literature regarding the immediate placement of dental implants, particularly in terms of predictability and survival rate, patient satisfaction with this surgical approach represents a highly relevant aspect (2,3). The primary advantage of immediate implant placement following tooth extraction lies in the execution of a single surgical procedure, which leads to a reduced overall treatment time, lower morbidity, and decreased postoperative discomfort, as well as the possibility of prosthetic rehabilitation through immediate loading in selected cases (5).

An increase in the number of publications on immediate implants in the European continent was observed. In this top 100, Europe had the highest number of publications (61%). Despite the significant number of studies from the European continent, the United States was the country with the most publications, accounting for 20% of the total studies included. Countries such as the United States and China have governments and foundations with significant influence in funding medical and dental research. However, the funding agencies linked to the studies included in this review were located in Sweden (Astra Tech AB) and Switzerland (Nobel Biocare, Thommen Medical and Osteology Foundation). This may explain the prominent position of the European continent, since investments, regardless of their source, are crucial means to foster and develop technology in various fields (22,23).

Among the universities highlighted in this review, three of them—the University of Bern (Switzerland), the Ghent University (Belgium), and the University of Zurich (Switzerland) are located in Europe. Among the authors of particular prominence, both Buser D and Lang NP were affiliated with universities in the European continent, specifically the University of Bern. Both authors have held prominent positions in the fields of periodontics and implantology worldwide.

Limitations of this study include the use of only the WoS-CC database, as there are other databases available for scientific research, such as Scopus, Medline, and Google Scholar. The decision to use only WoS-CC was based on other important bibliometric analyses in dentistry, as it allows sorting articles based on the number of citations (13,16,18) and is one of the main databases recommended for this type of study (14). Despite these limitations, the data presented in this study still provide a significant insight into the achievements and evolution of trends in the use of bone grafts in dentistry over the years.

## Conclusions

The conclusion drawn from the analysis of the 100 most-cited articles related to immediate dental implant placement indicates that they are primarily interventional studies, predominantly published in the United States and Switzerland, and featured in the Clinical Oral Implants Research journal.

## Figures and Tables

**Table 1:** List of journals among the 100 most-cited articles.

Journal Title	Number of Articles	Number of Citations	Impact Factor
Clinical Oral Implants Research	30	5,172	4.8
International Journal of Oral & Maxillofacial Implants	23	3,892	1.7
Journal of Periodontology	18	2,882	4.2
Journal of Clinical Periodontology	10	1,862	5.8
Clinical Implant Dentistry and Related Research	6	517	3.7
International Journal of Periodontics & Restorative Dentistry	5	616	1.3
Journal of Dental Research	2	288	5.7
Oral Surgery Oral Medicine Oral Pathology Oral Radiology and Endodontology	1	197	-
European Journal of Oral Implantology	1	176	-
Journal of Oral and Maxillofacial Surgery	1	141	2.3
Cochrane Database of Systematic Reviews	1	110	8.8
Journal of Oral Rehabilitation	1	106	3.1
Periodontology 2000	1	104	17.5

**Table 2:** Top 10 institutions with the highest number of articles among the top 100 most-cited.

Institution	Country	Number of articles	Number of citations
University of Bern	Switzerland	10	1,371
Loma Linda University	USA	8	1,236
University Melbourne	Australia	6	1,691
University of Ghent	Belgium	6	787
University Zurich	Switzerland	5	636
State University of Maringá	Brazil	4	1,007
Aarhus University	Denmark	4	467
New York University	USA	4	424
University of Gothenburg	Sweden	3	866
Free University Brussels	Belgium	3	476

**Table 3:** Top 10 authors with the highest number of publications among the most-cited articles.

Authors	Number of articles among the 100 most-cited	Number of citations among the most-cited articles
Buser D	11	2,311
Lang NP	11	1,894
Cosyn JAN	10	1,409
Lindhe J	9	2,151
Chen ST	9	2,402
De Bruyn H	8	963
Belser UC	8	1,524
Kan JYK	8	1,236
Rungcharassaeng K	7	1,132
De Rouck T	5	804

## Data Availability

The datasets used and/or analyzed during the current study are available from the corresponding author.
